# Assessing the mortality burden after acute myocardial infarction in SLE: insights from the MINAP Registry

**DOI:** 10.1093/rheumatology/keag365

**Published:** 2026-07-11

**Authors:** Kian Kermani, Andrew Cole, Nicholas Weight, Muhammad Rashid, Mamas A Mamas

**Affiliations:** Keele Cardiovascular Research Group, Centre for Prognosis Research, Institute for Primary Care and Health Sciences, Keele University, Stoke-on-Trent, UK; Keele Cardiovascular Research Group, Centre for Prognosis Research, Institute for Primary Care and Health Sciences, Keele University, Stoke-on-Trent, UK; Keele Cardiovascular Research Group, Centre for Prognosis Research, Institute for Primary Care and Health Sciences, Keele University, Stoke-on-Trent, UK; Keele Cardiovascular Research Group, Centre for Prognosis Research, Institute for Primary Care and Health Sciences, Keele University, Stoke-on-Trent, UK; Department of Cardiovascular Sciences, University of Leicester, Leicester, UK; National Institute for Health Research (NIHR) Leicester Cardiovascular Biomedical Research Unit, University of Leicester, Glenfield Hospital, Leicester, UK; Keele Cardiovascular Research Group, Centre for Prognosis Research, Institute for Primary Care and Health Sciences, Keele University, Stoke-on-Trent, UK; National Institute for Health and Care Research (NIHR) Birmingham Biomedical Research Centre, Institute of Translational Medicine, Birmingham, UK

**Keywords:** SLE, acute myocardial infarction, cardiovascular mortality

## Abstract

**Objectives:**

Acute myocardial infarction (AMI) is a leading cause of mortality globally. Although SLE is known to increase the risk of AMI, its impact on long-term outcomes following AMI in contemporary populations remains poorly defined. To determine whether individuals with SLE experience higher long-term mortality after AMI compared with those without SLE.

**Methods:**

Adults hospitalized with AMI between January 2005 and March 2019 were identified from the Myocardial Ischaemia National Audit Project registry and linked with Hospital Episode Statistics and Office for National Statistics mortality records. Long-term all-cause and cardiovascular mortality were assessed up to 5 years post-AMI.

**Results:**

Of 784 091 patients with AMI, 715 (0.1%) had a diagnosis of SLE. Patients with SLE were younger (median 63.9 vs 70.3 years; *P* < 0.001) and more often female (76% vs 34%; *P* < 0.001). Adjusted all-cause mortality was higher among those with SLE from 30 days (HR 1.62; 95% CI 1.23, 2.12; *P* = 0.001) to 5 years (HR 1.84; 95% CI 1.63, 2.09; *P* < 0.001). Adjusted cardiovascular mortality was similarly elevated at 30 days (HR 1.63; 95% CI 1.20–2.21; *P* = 0.002) and 5 years (HR 1.75; 95% CI 1.46–2.10; *P* < 0.001).

**Conclusions:**

Individuals with SLE have a persistently higher risk of all-cause and cardiovascular mortality following AMI, despite being younger and having less traditional cardiovascular comorbidities. These findings highlight SLE as an independent determinant of adverse post-AMI outcomes and support aggressive, guideline-directed secondary prevention in this high-risk population.

## Graphical abstract

**Graphical Abstract:**
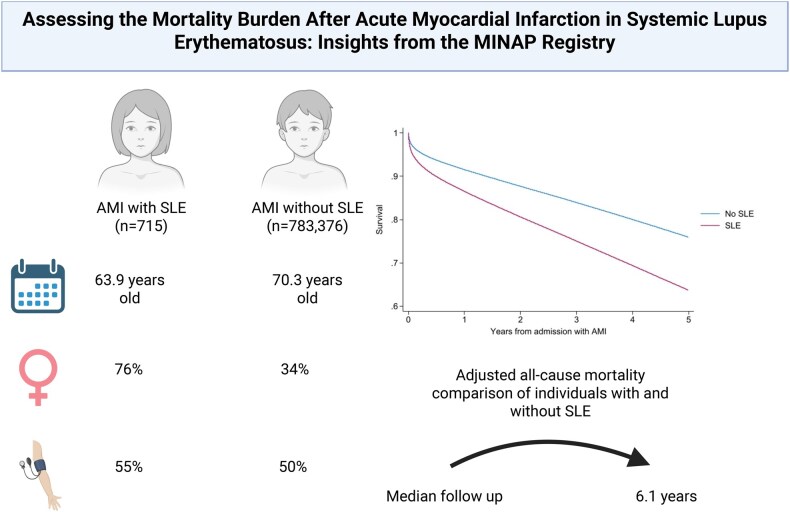

Rheumatology key messages

- Patients with SLE face persistently elevated mortality after AMI despite younger age and fewer comorbidities.
- SLE independently predicts worse long-term cardiovascular outcomes, highlighting inflammation-driven risk beyond traditional factors alone.
- Optimizing secondary prevention and multidisciplinary care is essential to reduce excess post-AMI mortality in SLE populations.

## Introduction

SLE is a chronic, multisystem, autoimmune condition associated with substantial cardiovascular morbidity [1]. Individuals with SLE have an estimated 5–6-fold higher risk of acute myocardial infarction (AMI) compared with the general population [2]. Although AMI represents a leading cause of death globally [3], evidence on long-term outcomes following AMI, within individuals with SLE, remains limited [4]. Existing studies are constrained by small sample sizes and short follow-up durations owing to the relative rarity of SLE.

Most prior studies have focused on short-term or in-hospital outcomes, with inconsistent results. In an analysis of the US Nationwide Inpatient Sample (2005–2014), Ando *et al.* reported comparable inpatient mortality following AMI between patients with and without SLE [5]. Whereas Sagheer *et al.* using the 2018 US Nationwide Readmissions Database, observed higher inpatient mortality and 30-day readmission rates among those with SLE [6]. However, no studies have examined long-term mortality beyond the index admission.

Using data from the Myocardial Ischaemia National Audit Project (MINAP) registry, linked with the Office for National Statistics (ONS) mortality records and Hospital Episode Statistics (HES), we compared long term all-cause and cardiovascular mortality up to 5 years after AMI in patients with and without SLE, and assessed differences in inpatient AMI care between the two groups.

## Methods

### Study design

This was a retrospective cohort study of prospectively collected data from the MINAP linked to ONS and HES data. MINAP is a prospective national registry of patients with acute coronary syndrome (ACS) admitted to 230 hospitals in England and Wales. MINAP includes data on patient demographics, clinical characteristics, comorbidities, pharmacotherapy, management and in-hospital outcomes, on ∼90 000 patients admitted with ACS per year. HES is a national registry covering all admissions to National Health Service Hospitals in England [7]. Data are recorded at the person level as a consultant episode for admitted patients and >17 million consultant episodes are added yearly. Each HES record contains clinical information including diagnosis and comorbidities. ONS is the independent provider of mortality statistics in the United Kingdom (UK), collecting data on all deaths registered in England and Wales using *International Classification of Diseases, Tenth Revision (ICD-10)* codes, and cause of death from the Medical Certificate of Cause of Death. Mortality follow-up was available for all included patients up to 31 July 2021.

### Study population

All index admissions between January 2005 and March 2019 with a primary diagnosis of AMI (comprising both ST-elevation myocardial infarction (STEMI) and non-ST segment myocardial infarction (NSTEMI)) were extracted from MINAP and stratified according to the presence of SLE at the time of presentation using ICD-10 codes (Supplementary Table S1). We elected not to include drug-induced SLE as part of the SLE cohort. AMI diagnosis was made by clinicians according to the presenting history, clinical examination and investigations, and the results of inpatient investigations in keeping with the consensus document of the Joint European Society of Cardiology (ESC) and American College of Cardiology (ACC) [8]. Patients were excluded if there were missing data for variables including in-hospital mortality, MACE, cause of death or missing unique identifier, which is the patient’s National Health Service (NHS) number. The first admission with AMI over our study period for each patient was included, with duplicate records or readmissions over the study period identified and removed using date of admission and NHS number.

### Outcomes

The primary outcomes were all-cause mortality, assessed at 30 days, 1 year, 5 years and over the entire study period, which ended in July 2021, which we referred to as “overall mortality”. Secondary outcomes of interest were quality of care measures, including the Opportunity Based Quality Indicators (OBQI), which consists of prescription of aspirin, P2Y12 inhibitors, ß-blockers, statins, ACE inhibitors/ARBs and whether referral to cardiac rehabilitation was made while an inpatient [9, 10].

### Statistical analysis

Continuous variables such as age at admission and BMI were summarized using mean and S.D. if normally distributed and median and interquartile ranges (IQR) if data were not normally distributed. Normality of distribution was assessed using the Shapiro–Wilk test. These data were compared using Student’s *t*-test if normally distributed, and Wilcoxon rank sum test if not normally distributed. Categorical variables were compared using the Pearson *χ*^2^ (*X*^2^) test and summarized as percentages (%). Multiple imputation by chained equations (MICE) with 10 imputed datasets and 100 iterations, was utilized to account for the missing data across our dataset. Our imputation model closely resembled our analysis model displayed below. MICE is the best practice when dealing with missing data and can provide unbiased estimates even with high levels of missingness, and some protection when data are missing, not at random [11]. Results of the complete case analysis undertaken prior to multiple imputation are shown in Supplementary Table S2. The proportion of missing data varied across variables included within the multiple imputation model; this is summarized along with the number of observations generated in Supplementary Fig. S1.

Multivariate Cox models were applied to 10 imputed datasets to generate adjusted hazard ratios (aHR) with 95% CIs for mortality over our study period, with estimates combined using Rubin’s rules [12]. Covariates were selected to include key baseline demographic features and relevant comorbidities, based on clinical consensus. Our model was adjusted for the following characteristics: age at admission, sex, ethnicity, year of admission, hospital region, heart rate, blood pressure, co-morbid conditions (hypertension, diabetes mellitus, history of asthma or chronic obstructive pulmonary disease, history of stroke or peripheral vascular disease, hypercholesterolaemia, family history of coronary artery disease, smoking history, chronic kidney disease (CKD), previous AMI, angina, previous PCI and previous CABG). Hazard ratios shown are from comparison of patients with SLE at time of admission with AMI to those without SLE. Separate Cox-models were used to create hazard ratios for 30-day, 1-year, 5-year and overall mortality (referring to the study end point of 31 July 2021). Kaplan–Meier curves were plotted to demonstrate unadjusted survival, and the stcurve function was used in Stata 18.0 to illustrate adjusted survival, using the previously specified Cox-model on a single extracted dataset from our imputed model. Cox-models were judged to be appropriate after assessment of Kaplan–Meier survival curves and confirmed by the assessment of the Schoenfeld residuals, demonstrating proportional hazard over our study period. Kaplan–Meier and log–log plots are shown in Supplementary Figs S2 and S3. A secondary analysis was undertaken to assess risk of cardiovascular mortality over the study period, adjusting for the same variable list as the previous Cox-model, with cardiovascular mortality as the failure, and remaining non-cardiovascular mortality censored at the time of occurrence. Sensitivity analyses were undertaken using a ‘1:1’ propensity score matched population, using the ‘psmatch2’ function on Stata 18.0. A total of 708 patients with SLE were matched to 708 patients without SLE in the propensity score-matched cohort. Patients were matched for the covariates included in the previously described Cox-model. Covariate balance before and after matching was assessed using standardized mean differences and Rubin’s balance diagnostics generated using the ‘pstest’ command in Stata. The same adjusted Cox regression model was subsequently applied to the matched population. A Fine and Gray competing risk regression model was fitted to the matched population, adjusting for the same covariates, with cardiovascular mortality as the outcome of interest, and non-cardiovascular mortality as the competing risk.

All statistical analyses performed using the Stata version 18.0 (StataCorp, College Station, TX, USA).

### Ethics

Secondary use of anonymized MINAP dataset for research purposes is authorized under NHS research governance arrangements and further supported under section 251 of NHS act 2006 (NIGB: ECC1-06(d)/2011), which allows researchers to use patient information collected within the dataset for medical research without patient consent. Therefore, a formal ethical approval was not sought for this study. Approval of the linkage of MINAP, HES and ONS registries, all UK-based institutions, was granted by the Health and Care Research Wales and the Health Research Authority (Research Ethics Committee reference 20/WA/0312). Additionally, approval was obtained by the Confidentiality Advisory Group, an independent body providing expert advice on the use of confidential patient information for research. This study also used publicly available aggregated NHS Workforce Statistics published by NHS England. The dataset contains no identifiable personal information; therefore, research ethics committee approval and individual consent were not required.

## Results

After applying the relevant exclusion criteria, 784 091 patients admitted with AMI, between January 2005 and March 2019 were included (Supplementary Fig. S4). Of whom, 715 (0.1%) had a diagnosis of SLE. The median duration of follow-up was 6.1 years.

### Demographic comparison between individuals with and without a diagnosis of SLE

Compared with patients without SLE, those with SLE were younger (median age: 63.9, IQR 54.2–72.1 years vs 70.3, IQR 59.2–80.3 years) and more often female (76% vs 34%, *P* < 0.001) (Table 1). They were less likely to be of White ethnicity (86% vs 91%, *P* < 0.001) and more likely to be of Asian (8% vs 6%, *P* < 0.001) or Black ethnicities (3% vs 1%, *P* < 0.001).

**Table 1:** Baseline demographics, management strategy and clinical outcome of individuals with AMI according to the presence of SLE.

Variables	AMI with SLE (*n* = 715)	AMI with no SLE (*n* = 783 376)	*P*-value
Age, years, median (IQR)	63.9 (54.2–72.1)	70.3 (59.2–80.3)	<0.001
Female (%)	545/715 (76)	264 572/783 376 (34)	<0.001
BMI, median [IQR]	26.2 (23.1–30.5)	26.9 (24.0–30.5)	0.031
Ethnicity			
White (%)	370/432 (86)	357 035/393 766 (91)	<0.001
Asian (%)	33/432 (8)	24 176/393 766 (6)	
Black (%)	15/432 (3)	3940/393 766 (1)	
Mixed (%)	3/432 (1)	914/393 766 0	
Other (%)	11/432 (3)	7701/393 766 (2)	
Killip class			
Basal crepitations (%)	66/381 (17)	44 767/349 151 (13)	0.067
Pulmonary oedema (%)	16/381 (4)	17 510/349 151 (5)	
Cardiogenic shock (%)	6/381 (2)	5757/349 151 (2)	
ECG ST changes (%)	562/690 (81)	657 627/757 541 (87)	<0.001
Previous smoker (%)	178/660 (27)	241 521/724 968 (33)	0.002
Current smoker (%)	193/660 (29)	203 337/724 968 (28)	
CCF (%)	31/642 (5)	37 618/709 720 (5)	0.594
Hypercholesterolemia (%)	171/644 (27)	224 427/706 863 (32)	0.005
Cerebrovascular disease (%)	68/643 (11)	58 789/709 976 (8)	0.035
History of angina (%)	151/647 (23)	161 789/715 879 (23)	0.654
Peripheral vascular disease (%)	41/638 (6)	30 511/703 965 (4)	0.010
Chronic kidney disease^a^ (%)	75/643 (12)	39 774/708 747 (6)	<0.001
Diabetes mellitus (%)	112/695 (16)	157 562/756 397 (21)	0.002
Hypertension (%)	354/649 (55)	359 773/722 943 (50)	0.015
Asthma/COPD (%)	114/636 (18)	108 182/706 234 (15)	0.068
Family history of CAD (%)	150/515 (29)	179 704/585 869 (31)	0.447
Previous AMI (%)	119/654 (18)	124 629/723 060 (17)	0.516
Previous PCI (%)	59/641 (9)	50 497/711 501 (7)	0.038
Previous CABG (%)	28/643 (4)	39 991/712 890 (6)	0.167
STEMI (%)	235/715 (33)	305 738/783 376 (39)	0.001
Heart rate, bpm, median (IQR)	80 (69–94)	78 (66–92)	0.045
Systolic blood pressure, median (IQR)	135 (117–156)	138 (120–157)	0.056
LV function^b^			
Good (%)	223/516 (45)	194 218/543 850 (36)	<0.001
Moderate (%)	98/516 (19)	119 816/543 850 (22)	
Severe (%)	34/516 (7)	39 723/543 850 (7)	
LV function not assessed (%)	151/516 (29)	190 093/543 850 (35)	
Cardiac arrest (%)	30 700 (4)	48 397/758 693 (6)	0.023
Warfarin (%)	65/582 (11)	33 789/639 083 (5)	<0.001
Glycoprotein 2b/3a inhibitor (%)	40/585 (7)	56 281/651 552 (9)	0.121
IV Nitrate (%)	102/577 (18)	113 042/639 599 (18)	0.998
MRA (%)	35/448 (8)	34 254/457 891 (7)	0.790
Aspirin (%)	661/705 (94)	748 776/777 120 (96)	<0.001
P2Y12 inhibitor (%)	592/697 (85)	641 583/750 229 (86)	0.662
Statins (%)	552/702 (79)	638 814/772 428 (83)	0.004
ACE inhibitors/ARB (%)	499/700 (71)	574 880/769 784 (75)	0.039
Beta-blockers (%)	538/700 (77)	607 513/771 529 (79)	0.223
Inpatient invasive coronary angiogram (%)	481/674 (71)	508 866/743 284 (68)	0.105
Inpatient percutaneous coronary intervention (%)	311/705 (44)	334 388/768 060 (44)	0.758
CABG surgery (%)	11/519 (2)	16 881/601 428 (3)	0.343
Revascularization (CABG surgery/PCI) (%)	322/705 (46)	350 472/768 060 (46)	0.982
Inpatient mortality (%)	44/715 (6)	46 088/783 376 (6)	0.759
Thirty-day mortality (%)	53/715 (7)	56 105/783 376 (7)	0.795
1-year mortality (%)	124/715 (17)	124 717/783 376 (16)	0.299
5-year all-cause mortality (%) (Kaplan–Meier estimate)	37%	34%	
5-year cardiovascular mortality (%) (Kaplan–Meier estimate)	17%	17%	
Reinfarction (%)	10/653 (2)	10 225/705 476 (1)	0.861
Major bleeding (%)	3/692 0	6568/753 082 (1)	0.215
MACE^c^ (%)	50/692 (7)	51 654/753 082 (7)	0.703

Medication usage reflects assessment of medications at the point of discharge.

Continuous variables are expressed as median (IQR) and categorical variables as proportions (%). Denominators represent the total number of participants with a data point collected; numerators represent the number of those participants for whom the variable of interest was present. Cardiac arrest is a composite of both in-hospital and out of hospital cardiac arrests.

Chronic kidney disease is recorded in the MINAP registry as a serum creatinine level chronically elevated above 200 µmol/l.

a Good left ventricular function was defined as an ejection fraction (EF) ≥50%, moderate LV function as an EF 30–49% and severe LV function as an EF <30%.

b Chronic kidney disease is recorded in the MINAP registry as a serum creatinine level chronically elevated above 200 µmol/l.

c MACE is defined as composite end point of in-hospital death and reinfarction.

ACE: angiotensin-converting enzyme, AMI: acute myocardial infarction, ARB: angiotensin receptor blockers, bpm: beats per min, CABG: coronary artery bypass graft, CCF: congestive cardiac failure, COPD: chronic obstructive pulmonary disease, IQR: interquartile range, IV: intravenous, LV: left ventricular, MACE: major adverse cardiovascular events, MRA: mineralocorticoid receptor antagonist, PCI: percutaneous coronary intervention.

Individuals with SLE less frequently had STEMI presentations (33% vs 39%, *P* = 0.001) with comorbidities such as CKD (12% vs 6%; *P* < 0.001) and hypertension (55% vs 50%; *P* = 0.015) more prevalent among those with SLE, whereas hypercholesterolaemia (27% vs 32%; *P* = 0.005) and diabetes mellitus (16% vs 21%; *P* = 0.002) were less common (Table 1).

### Management strategies and clinical outcomes for individuals with and without SLE

Rates of inpatient coronary angiography were similar (71% vs 68%, *P* = 0.105), as were overall revascularization rates by PCI or CABG (46% vs 46%, *P* = 0.982) (Table 1). Among those with NSTEMI, individuals with SLE were less likely to undergo coronary angiography within 72 h (53% vs 57%; *P* = 0.013).

At discharge, patients with SLE were less likely to receive aspirin (94% vs 96%, *P* < 0.001), statins (79% vs 83%, *P* = 0.004) and ACE inhibitor/ARB therapy (71% vs 75%, *P* = 0.039) (Table 1). Overall quality of care was broadly similar, although the mean OBQI score was slightly lower in the SLE group (81.1 vs 82.7, *P* = 0.032) (Supplementary Table S3).

**Figure 1:**
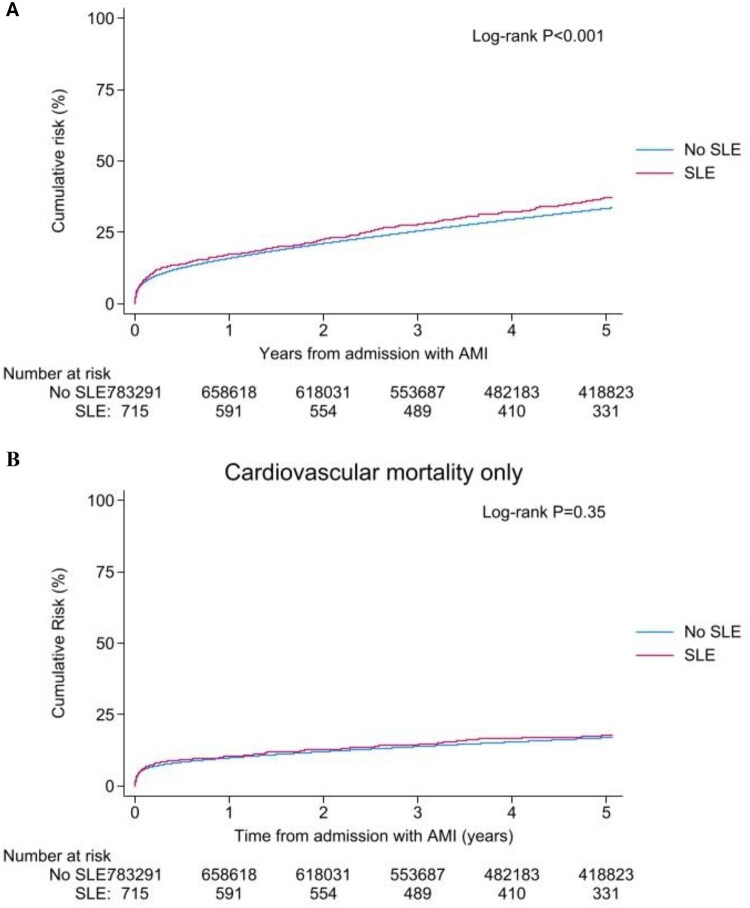
Kaplan–Meier charts for mortality comparison of individuals with SLE and without. (**A**) All-cause mortality. (**B**) Cardiovascular mortality

Unadjusted all-cause mortality at 30 days (7% vs 7%; *P* = 0.795) and 1 year (17% vs 16%; *P* = 0.299) did not differ significantly between individuals with and without SLE (Table 1; Fig. 1). By 5 years, Kaplan–Meier estimates suggested higher all-cause mortality in the SLE cohort (37% vs 34%), whereas the estimated 5-year cardiovascular mortality was similar (17% vs 17%) (Fig. 1).

**Figure 2:**
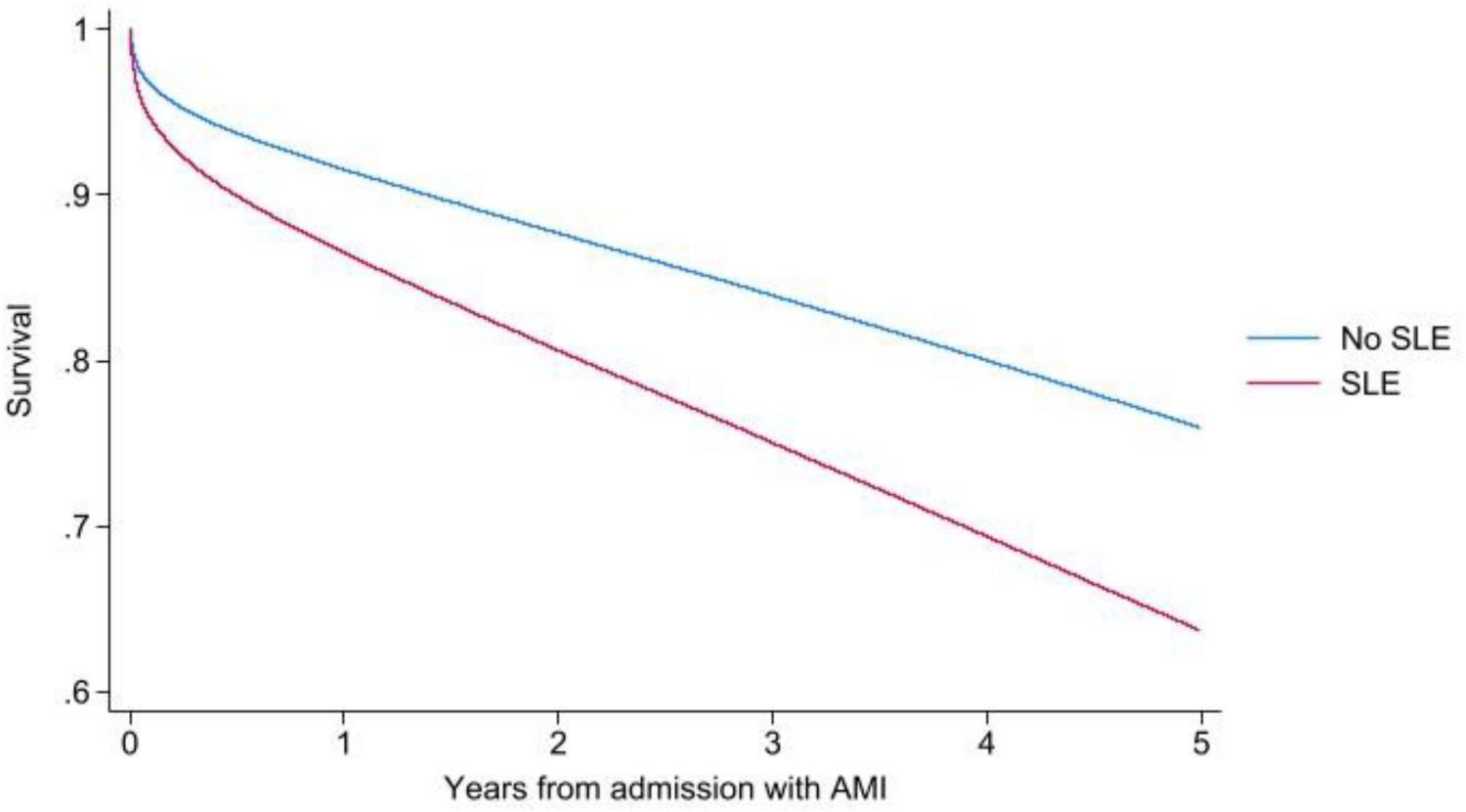
Adjusted all-cause mortality comparison of individuals with SLE and without. Adjusted hazard ratios are presented with 95% CIs, adjusted for: age at admission, sex, ethnicity, year of admission, heart rate, blood pressure, co-morbid conditions (hypertension, diabetes mellitus, history of asthma or COPD, history of CVA or PVD, hypercholesterolaemia, family history of coronary artery disease, smoking history, chronic renal failure, previous AMI, angina, previous PCI and previous CABG) and admission hospital

After multivariable adjustment, however, SLE was independently associated with higher all-cause mortality at 30 days (adjusted hazard ratio [aHR] 1.62; 95% CI 1.23, 2.12; *P* = 0.001), 1 year (aHR 1.77; 95% CI 1.48, 2.11; *P* < 0.001) and 5 years (aHR 1.84; 95% CI 1.63, 2.09; *P* < 0.001) (Table 2; Fig. 2). Similarly, SLE was associated with higher cardiovascular mortality at 30 days (aHR 1.63; 95% CI 1.20, 2.21; *P* = 0.002), 1 year (aHR 1.73; 95% CI 1.38, 2.19; *P* < 0.001) and 5 years (aHR 1.75; 95% CI 1.46–2.10; *P* < 0.001) (Table 2). Similar findings were obtained, within a 1:1 propensity score matched population (Table 3).

**Table 2:** Survival analysis including both all-cause and cardiovascular mortality for individuals with AMI with or without SLE.

Outcome variables	Adjusted hazard ratio for individuals with SLE compared those without (95% CIs)	*P*-value
Primary outcomes: all-cause mortality		
30-day mortality	1.62 (1.23–2.12)	0.001
1-year mortality	1.77 (1.48–2.11)	<0.001
5-year mortality	1.84 (1.63–2.09)	<0.001
Overall mortality	1.84 (1.66–2.05)	<0.001
Primary outcomes: cardiovascular mortality		
30-day mortality	1.63 (1.20–2.21)	0.002
1-year mortality	1.73 (1.38–2.19)	<0.001
5-year mortality	1.75 (1.46–2.10)	<0.001
Overall mortality	1.72 (1.45–2.04)	<0.001

Adjusted hazard ratios are presented with 95% CIs, adjusted for: age at admission, sex, ethnicity, year of admission, heart rate, blood pressure, co-morbid conditions (hypertension, diabetes mellitus, history of asthma or COPD, history of CVA or PVD, hypercholesterolaemia, family history of coronary artery disease, smoking history, chronic renal failure, previous AMI, angina, previous PCI and previous CABG) and admission hospital.

Non-CV mortality censored at time of occurrence.

**Table 3:** 1:1 propensity score matched population with SLE and without for all-cause mortality.

Outcome variables	Adjusted hazard ratio for individuals with SLE compared those without (95% CIs)	*P*-value
Primary outcomes		
30-day mortality	1.66 (1.06–2.59)	0.026
1-year mortality	1.47 (1.11–1.94)	0.007
5-year mortality	1.61 (1.32–1.96)	<0.001
Overall mortality	1.63 (1.37–1.93)	<0.001

A total of 708 patients with SLE were matched to 708 patients without SLE using 1:1 propensity score matching. Covariate balance before and after matching was assessed using standardized mean differences and Rubin’s balance diagnostics generated using the pstest command in Stata. Following matching, mean standardized bias reduced from 15.1% to 2.6%, with Rubin’s *B* decreasing from 125.4–15.4 and Rubin’s *R* of 0.96, consistent with acceptable post-match covariate balance. Adjusted hazard ratios are presented with 95% CIs, adjusted for age at admission, sex, ethnicity, year of admission, heart rate, blood pressure, co-morbid conditions (hypertension, diabetes mellitus, history of asthma or COPD, history of CVA or PVD, hypercholesterolaemia, family history of coronary artery disease, smoking history, chronic kidney disease, previous AMI, angina, previous PCI and previous CABG) and admission hospital.

Cardiovascular causes accounted for the majority of deaths in both the groups (Supplementary Fig. S4). In competing-risk regression analyses, SLE was associated with higher sub-distribution hazards of cardiovascular mortality than those without SLE at 1 year (sHR 1.47, 95% CI 1.01, 2.11, *P* = 0.039), 5 years (sHR 1.60, 95% CI 1.20, 2.15, *P* = 0.001) and across overall follow-up (sHR 1.55, 95% CI 1.19, 2.02, *P* = 0.001), whereas the association at 30 days did not reach statistical significance (sHR 1.59, 95% CI 0.94, 2.55, *P* = 0.084) (Table 4).

**Table 4:** Competing risk regression analysis of cardiovascular mortality survival analysis for individuals with AMI with or without SLE.

Outcome variables	Adjusted sub-hazard ratio for individuals with SLE compared those without (95% CIs)	*P*-value
Primary outcomes		
30-day mortality	1.59 (0.94–2.55)	0.084
1-year mortality	1.47 (1.01–2.11)	0.039
5-year mortality	1.60 (1.20–2.15)	0.001
Overall mortality	1.55 (1.19–2.02)	0.001

Adjusted hazard ratios are presented with 95% CIs, adjusted for: age at admission, sex, ethnicity, year of admission, heart rate, blood pressure, co-morbid conditions (hypertension, diabetes mellitus, history of asthma or COPD, history of CVA or PVD, hypercholesterolaemia, family history of coronary artery disease, smoking history, chronic renal failure, previous AMI, angina, previous PCI and previous CABG) and admission hospital. Non-cardiovascular mortality is included as a competing risk in a Fine and Gray competing risk regression model.

## Discussion

In this contemporary national cohort of over 780 000 patients admitted with AMI, we found that individuals with SLE experienced significantly higher adjusted risks of both all-cause and cardiovascular mortality extending up to 5 years post-AMI. This excess risk persisted despite patients with SLE being younger and having fewer traditional cardiovascular risk factors compared with those without SLE.

SLE is a chronic autoimmune disease characterized by systemic inflammation and immune-mediated injury [13] affecting multiple organs, including the cardiovascular system [14]. Cardiac involvement may include pericardial and myocardial inflammation, conduction abnormalities, valve disease and coronary artery pathology [15]. It is well established that SLE accelerates atherosclerosis [16], predisposing individuals to premature coronary artery disease and an increased risk of thrombotic occlusion leading to AMI [17]. Previous studies have reported up to a 6-fold greater risk compared with the general population [2, 18]. Persistent inflammation, endothelial dysfunction and immune-mediated vascular injury likely continue to drive atherothrombosis and adverse myocardial remodelling after the acute event, contributing to the observed excess long-term mortality [19].

Despite this elevated cardiovascular risk, individuals with SLE in our cohort had lower prevalences of several traditional comorbidities, including diabetes mellitus and hypercholesterolaemia. This may partly reflect the substantially younger age of patients with SLE at AMI presentation, as the prevalence of these traditional cardiovascular risk factors generally increases with age. This paradox may also reflect underdiagnosis, metabolic alterations from chronic disease or treatment effects from immunosuppressive therapy. These findings highlight that conventional cardiovascular risk factors alone cannot explain the heightened cardiovascular burden in SLE, underscoring the central role of inflammation, immune dysregulation and vascular injury.

It is therefore concerning that patients with SLE in our study were less likely to receive statins and ACE inhibitors despite clear evidence supporting their use following AMI [20, 21]. Similar disparities in the delivery of evidence-based cardiovascular care and long-term outcomes have also been demonstrated in other high-risk inflammatory and chronic disease populations [22, 23]. Although treatment decisions may have been influenced by CKD or concerns over polypharmacy, withholding evidence-based therapies should be exceptional [24]. Given the multisystem complexity of SLE and frequent use of immunomodulatory therapies, early multidisciplinary involvement, particularly with rheumatology, may help optimize long-term cardiovascular outcomes [25].

While SLE is well recognized as a major cardiovascular risk condition, evidence on outcomes following AMI remains limited and inconsistent. Previous studies have been restricted to short-term or inpatient outcomes. For example, Sagheer *et al.* reported higher inpatient mortality and 30-day readmission among SLE patients with AMI in the US Nationwide Readmissions Database (aOR = 1.40; 95% CI 1.1, 1.79, *P* = 0.006) [6]. Equally Ke *et al.* using the Taiwan National Health Insurance Database demonstrated similar findings of worse in-hospital mortality for individuals with SLE (OR 1.98; 95% CI: 1.2, 3.26) [26]. Lai *et al.* reported an even greater excess risk of in-hospital mortality in patients with SLE after AMI (OR 2.31; 95% CI 1.62–3.28) [27]. Our study is the first to evaluate long-term AMI outcomes in a large, contemporary national cohort and to demonstrate persistently higher adjusted mortality extending up to 5 years post-AMI.

This sustained excess mortality beyond hospital discharge likely reflects a multifactorial interplay between immune-mediated vascular injury, accelerated atherosclerosis and the limitations of current secondary prevention in addressing inflammation-driven cardiovascular risk. Without concurrent control of SLE activity patients may remain vulnerable to recurrent events [8]. Standard antiplatelet and anticoagulant regimens may also be suboptimal in this prothrombotic population [28]. In addition, procedural challenges, diffuse multivessel coronary disease [29], particularly in those with CKD, may limit the success of revascularization and contribute to poorer long-term outcomes [30]. Chronic corticosteroids use and other immunosuppressive therapies may further exacerbate cardiovascular risk [31]. Moreover, the predominance of women within the SLE cohort may partly explain the higher mortality, as women have been shown to receive less invasive management and to experience poorer AMI outcomes overall [32]. The higher proportion of Asian and Black patients with SLE in our study also raises the possibility that ethnic disparities in cardiovascular outcomes and access to care may contribute to the mortality gap [33].

Collectively, these findings highlight the need to recognize SLE as an independent determinant of long-term cardiovascular prognosis after AMI. Early identification of high-risk patients, optimization of secondary prevention and close collaboration between cardiology and rheumatology teams should form the foundation of post-AMI care. Targeted strategies that address both traditional and inflammation-mediated risk pathways may be required to mitigate the persistently elevated mortality risk observed in this population.

### Strengths

To our knowledge, this is the largest contemporary study to evaluate the long-term survival following AMI in patients with SLE. The use of the national MINAP registry provides comprehensive, prospectively collected data on all AMI admissions across England and Wales. Linkage with the ONS mortality records ensures complete and accurate long-term follow-up, allowing robust assessment of both all-cause and cardiovascular mortality.

The national coverage of MINAP minimizes regional variation, enhancing generalizability to other publicly funded healthcare systems. The long median follow-up of 6.1 years enables evaluation of both early and late post-discharge outcomes and captures trends before the COVID-19 pandemic, avoiding confounding effects of altered care pathways during that period.

### Limitations

This study shares several limitations with other large national registry analyses. Although MINAP captures a broad range of clinical variables, it lacks detailed information on frailty, angiographic findings, treatment rationale and some comorbidities. MINAPs predefined variable definitions can limit granularity; for example, CKD is defined solely by creatinine >200 μmol/l, preventing subclassification by disease stage. Furthermore, there is no external validation of data inputs and as with all registry databases there is potential for data entry error or under-reporting.

Data are collected at the time of AMI, and information on SLE duration, disease activity and treatment regimens is unavailable; thus, we cannot evaluate the effect of SLE severity or treatment on outcomes. Whilst we assessed prescribed medication at the point of discharge our analysis of this disparity in prescriptions is limited as we lack data on why individuals did not receive medications or if these were appropriately ceased prior to discharge on clinical grounds. Despite adjustment for key confounders, residual confounding from unmeasured variables cannot be excluded, and causal inference should therefore be made with caution.

## Conclusion

In this nationwide study of >780 000 patients with AMI, individuals with SLE experienced significantly higher adjusted all-cause and cardiovascular mortality compared with those without SLE, despite being younger and having fewer traditional cardiovascular risk factors. This excess risk persisted well beyond the acute phase. These findings highlight SLE as an independent determinant of adverse long-term cardiovascular outcomes and underscore the importance of early recognition, rigorous secondary prevention and coordinated cardiology–rheumatology care to mitigate ongoing risk in this vulnerable population.

## Supplementary Material

keag365_Supplementary_Data

## Data Availability

The authors do not have authorization to share the data, but it can be accessed through contacting the National Institute for Cardiovascular Outcomes Research (NICOR) upon approval.
