# Supplementary material for: Assessing the mortality burden after acute myocardial infarction in SLE: insights from the MINAP Registry
Source: Rheumatology (Oxford). 2026 Jul 11;65(7):keag365. doi: 10.1093/rheumatology/keag365 (PMC13399779; doi:10.1093/rheumatology/keag365)
Supplement: keag365_Supplementary_Data [file keag365_supplementary_data.docx]

| **ICD-10 Codes used** |
| --- |
| **M32.0:** Drug-induced systemic lupus erythematosus |
| **M32.1:** SLE with organ or system involvement |
| **M32.8:** Other forms of SLE |
| **M32.9:** SLE, unspecified |

**Supplementary Table S1: ICD-10 codes used to create SLE cohort**

| **Outcome variables** | **Adjusted hazard ratio for individuals with SLE compared those without (95% CIs)** | **P-value** |
| --- | --- | --- |
| **Primary Outcomes: All-cause mortality** | | |
| 30-day mortality | 1.32 (0.78-2.23) | 0.304 |
| 1-year mortality | 1.63 (1.20-2.23) | 0.002 |
| 5-year mortality | 1.88 (1.53-2.29) | <0.001 |
| Overall mortality | 1.93 (1.61-2.32) | <0.001 |
| **Primary Outcomes: Cardiovascular mortality** | | |
| 30-day mortality | 1.35 (0.75-2.43) | 0.324 |
| 1-year mortality | 1.58 (1.04-2.40) | 0.032 |
| 5-year mortality | 1.73 (1.26-2.37) | 0.001 |
| Overall mortality | 1.74 (1.30-2.34) | <0.001 |

**Supplementary Table S2: Complete case survival analysis including both all-cause and cardiovascular mortality for individuals with AMI with or without systemic lupus erythematosus (SLE).**

Results included for all patients with data for all variables included in survival models prior to multiple imputation.

Adjusted Hazard ratios are presented with 95% CIs, adjusted for: age at admission, sex, ethnicity, year of admission, heart rate, blood pressure, co-morbid conditions (hypertension, diabetes mellitus, history of asthma or COPD, history of CVA or PVD, hypercholesterolaemia, family history of coronary artery disease, smoking history, chronic renal failure, previous AMI, angina, previous PCI and previous CABG, and admission hospital.

|  | **NSTEMI and SLE (n=465)** | **NSTEMI with no SLE (n=460,889)** | **P-value** |
| --- | --- | --- | --- |
| Coronary Angiography received within 72 hours (%) | 107/201 (53) | 100,561/175,491 (57) | 0.013 |
| LV Function recorded in notes (%) | 240/347 (69) | 199,075/325,754 (61) | 0.002 |
| Fondaparinux or LMWH received (%) | 316/380 (83) | 341,077/387,432 (88) | 0.003 |
| DAPT received on discharge (%) | 370/457 (81) | 372,445/447,799 (83) | 0.207 |
| ACEi or ARB on discharge for those with moderate and severe LVSD (%) | 51/74 (69) | 58,872/77,471 (76) | 0.155 |
| Beta Blocker on discharge for those for those with moderate and severe LVSD (%) | 62/74 (84) | 63,646/77,587 (82) | 0.695 |
| Composite All/None score* (%) | 299/458 (65) | 312,931/448,042 (70) | 0.034 |
| Composite All/None score for those with moderate and severe LVSD (%) | 51/73 (70) | 56,684/77,315 (73) | 0.505 |
| Mean OBQI score | 81.1 | 82.7 | 0.032 |
| Cardiac rehabilitation (%) | 319/406 (79) | 326,661/418,316 (78) | 0.414 |

**Supplementary Table S3: Quality Indicators for individuals with NSTEMI according to presence of SLE (ESC ACVC and OBQI)**

ESC; European Society of Cardiology, ACVC; Association for Acute Cardiovascular Care, LV; left ventricle, EF; ejection fraction, LMWH; low molecular weight heparin, DAPT; dual antiplatelet therapy, ACEi/ARB; angiotensin converting enzyme inhibitor/angiotensin receptor blockers, LVSD; left ventricular systolic dysfunction, N/A; Not Available.

*Composite score of receipt of low dose aspirin, P2Y_12_ inhibition and statin.

**Opportunity based QI (The score consisted of 6 evidence-based processes of care: the prescription of aspirin, thienopyridine inhibitor, β-blocker, angiotensin converting enzyme inhibitor (ACEi), HMG CoA reductase enzyme inhibitor (statin) and enrolment onto a cardiac rehabilitation programme at the time of discharge). The OBCS reflects the number of care opportunities fulfilled at each hospital (numerator) divided by the number of opportunities to provide care (denominator). Excluded from both numerator and denominator were particular interventions that were contra-indicated, not applicable, not indicated in, or declined by, individual patients.

**
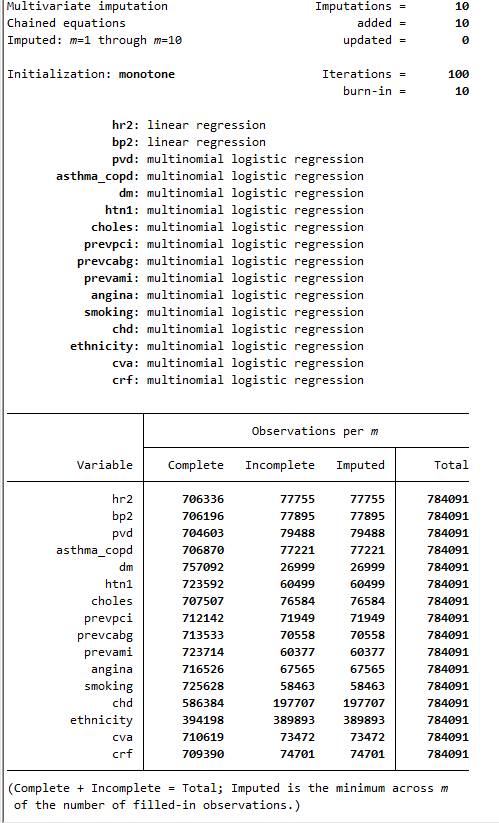
**

**Supplementary Figure S1: Multiple Imputation output with numbers of imputed observations for included variables in model**

**
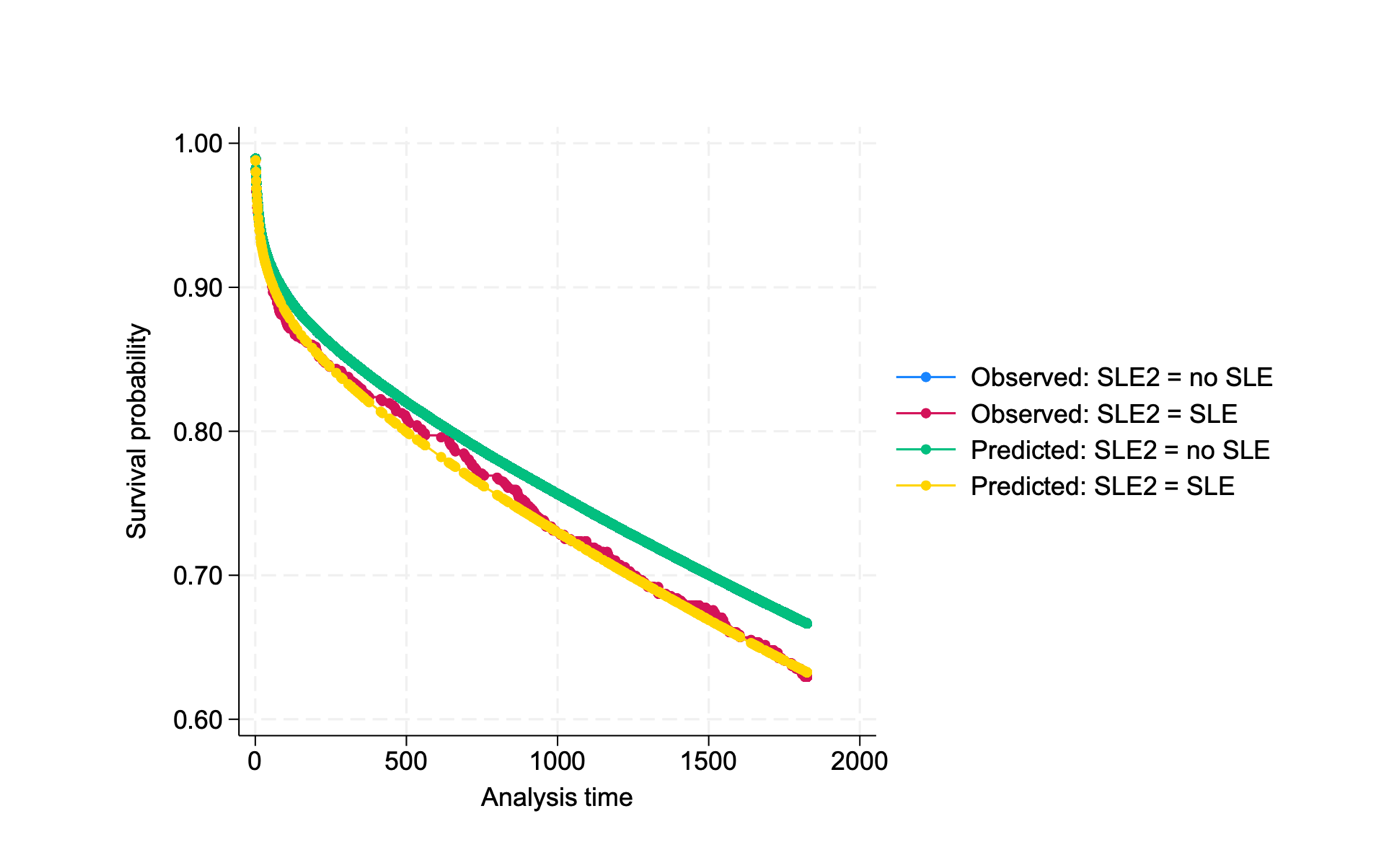
**

**Supplementary Figure S2: Proportional hazard model testing with comparison of Kaplan-Meier survival and survival from cox-model**

**
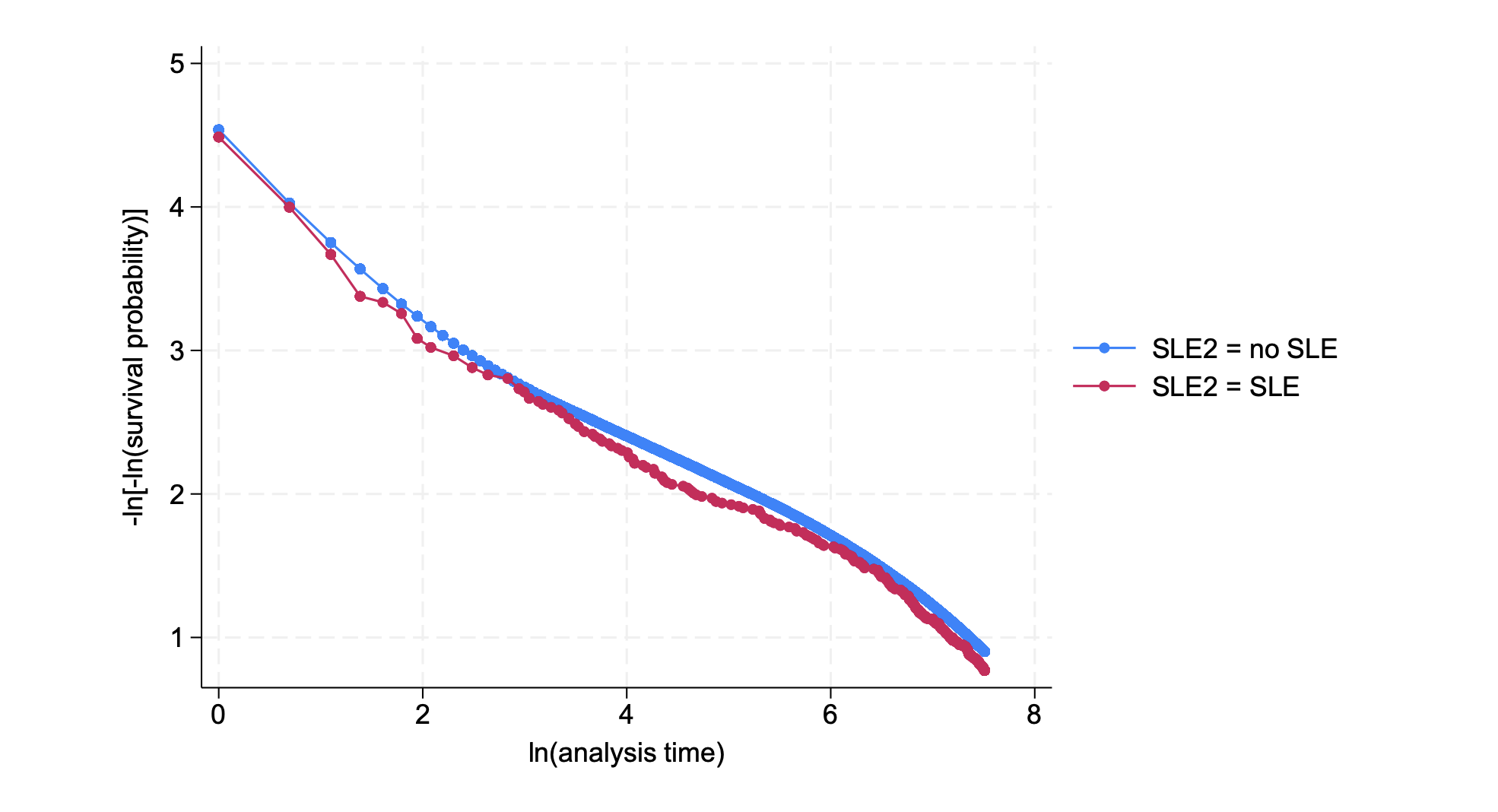
**

**Supplementary Figure S3: Proportional hazard model testing with log-log plot**

Total number of AMI patient episodes in HES MINAP ONS data from 2005 – 2019

(n=10,690,541)

Records excluded for missing cause of death

(n=57,838)

Records excluded for inconsistent mortality dates (n=8,881)

Records excluded if inconsistent admission or discharge dates (n=2,799,040)

Duplicate records excluded according to NHS number (n=7,040,691)

Total number of patients for inclusion

(n= 784,091)

Group 1: No SLE

(n=783,376)

Group 2: SLE

(n= 715)

**Supplementary Figure S4: STROBE diagram detailing exclusion criteria**
